# Relationship between the stroke mechanism of symptomatic middle cerebral artery atherosclerotic diseases and culprit plaques based on high-resolution vessel wall imaging

**DOI:** 10.3389/fneur.2022.968417

**Published:** 2022-09-16

**Authors:** Guo-hui Lin, Jian-xun Song, Teng-da Huang, Nian-xia Fu, Li-ling Zhong

**Affiliations:** Department of Radiology, Shenzhen Baoan People's Hospital, Shenzhen, China

**Keywords:** stroke, mechanism, atherosclerosis, middle cerebral artery, high-resolution vessel wall imaging

## Abstract

**Purpose:**

For patients with symptomatic middle cerebral artery (MCA) atherosclerotic stenosis, identifying the potential stroke mechanisms may contribute to secondary prevention. The purpose of the study is to explore the relationship between stroke mechanisms and the characteristics of culprit plaques in patients with atherosclerotic ischemic stroke in the M1 segment of the middle cerebral artery (MCA) based on high-resolution vessel wall imaging (HR-VWI).

**Methods:**

We recruited 61 patients with acute ischemic stroke due to MCA atherosclerotic stenosis from Shenzhen Bao'an District People's Hospital. According to prespecified criteria based on infarct topography and magnetic resonance angiography, possible stroke mechanisms were divided into parent artery atherosclerosis occluding penetrating artery (P), artery-to-artery embolism (A), hypoperfusion (H), and mixed mechanisms (M). The correlation between the characteristics of MCA M1 culprit plaque and different stroke mechanisms was analyzed using HR-VWI. The indicators included plaque surface irregularity, T1 hyperintensity, location, plaque burden (PB), remodeling index (RI), enhancement rate, and stenosis rate.

**Results:**

Parental artery atherosclerosis occluding penetrating artery was the most common mechanism (37.7%). The proposed criteria showed substantial to excellent interrater reproducibility (κ, 0.728; 0.593–0.863). Compared with the P group, the surface irregularity, T1 hyperintensity, and obvious enhancement of the culprit plaque in the A group were more common (*p* < 0.0125). Compared with the other stroke mechanisms, positive remodeling of culprit plaques was more common (*p* < 0.0125), the RI was greater (*p* < 0.05), and the PB was the smallest (*p* < 0.05) in the P group. The enhancement ratio (ER) was smaller in the P group (*p* < 0.05). Compared with the A group, T1 hyperintensity of the culprit plaque was more common in the H group (*p* < 0.0125), and the stenosis rate was greater (*p* < 0.05). After adjustment for clinical demographic factors in the binary logistic regression analysis, the enhancement level (odds ratio [*OR*] 0.213, 95% *CI* (0.05–0.91), *p* = 0.037) and PB of culprit plaque (*OR* 0, 95% *CI* (0–0.477), *p* = 0.034) were negatively associated with P groups.

**Conclusion:**

The culprit plaque characteristics of patients with symptomatic MCA atherosclerotic in different stroke mechanisms may be evaluated using HR-VWI. The plaque characteristics of different stroke mechanisms may have clinical value for the selection of treatment strategies and prevention of stroke recurrence.

**Clinical trial registration:**

Identifier: ChiCTR1900028533.

## Introduction

Intracranial large atherosclerosis is one of the most common causes of stroke worldwide (1). The vessel wall evolves from having a slight thickening to the development of a nonstenotic plaque, which gradually develops into lumen stenosis with significant hemodynamic changes and occlusion (2–4). This process often involves multiple arterial beds, and the middle cerebral artery is the most common. The incidence of the population is highest in Asia (5). The incidence rate of the intracranial large atherosclerotic disease has gradually increased in recent years with changes in the social environment, and it is affecting a younger population (6). Therefore, effective methods are needed to clarify the mechanism of ischemic stroke because these mechanisms have potential significance for the clinical treatment and prevention of secondary stroke (7).

The pathological changes of intracranial large artery atherosclerosis indicate that ischemic stroke may involve a variety of pathogeneses, including perforating artery occlusion due to maternal atherosclerosis, artery–artery embolism, hypoperfusion, and mixed mechanisms (8). There are differences in treatment options for these specific mechanisms. For example, single subcortical infarcts are treated with dual antithrombotic therapy, but the intravascular treatment of large atherosclerosis with significantly narrowed lumens remains controversial (9, 10). A recent study found that more patients with the baseline stroke mechanism of an artery–artery embolism + hypoperfusion had a history of dyslipidemia and hypertension than those with other stroke mechanisms (11). Dyslipidemia is a risk factor for the presence of vulnerable plaques, which may increase the risk of plaque rupture and subsequent A-A embolism. It has been reported that hypertension is associated with poor pial collateral circulation in patients with acute ischemic stroke (12), which may be a risk factor for the subtype mechanism of hypoperfusion stroke. On the other hand, current evidence suggests that enhanced contrast, positive remodeling, and plaque irregularity of intracranial plaques are associated with an increased risk of stroke (13). Therefore, clarifying the potential relationship between the stroke mechanism and plaque characteristics of large artery atherosclerosis may be valuable for individualized clinical treatment and patient management. The present study aimed to explore the relationship between stroke mechanisms and the characteristics of culprit plaques in patients with atherosclerotic ischemic stroke in the M1 segment of the middle cerebral artery (MCA) based on high-resolution vessel wall imaging (HR-VWI).

## Methods

### Patients

**Figure 1:**
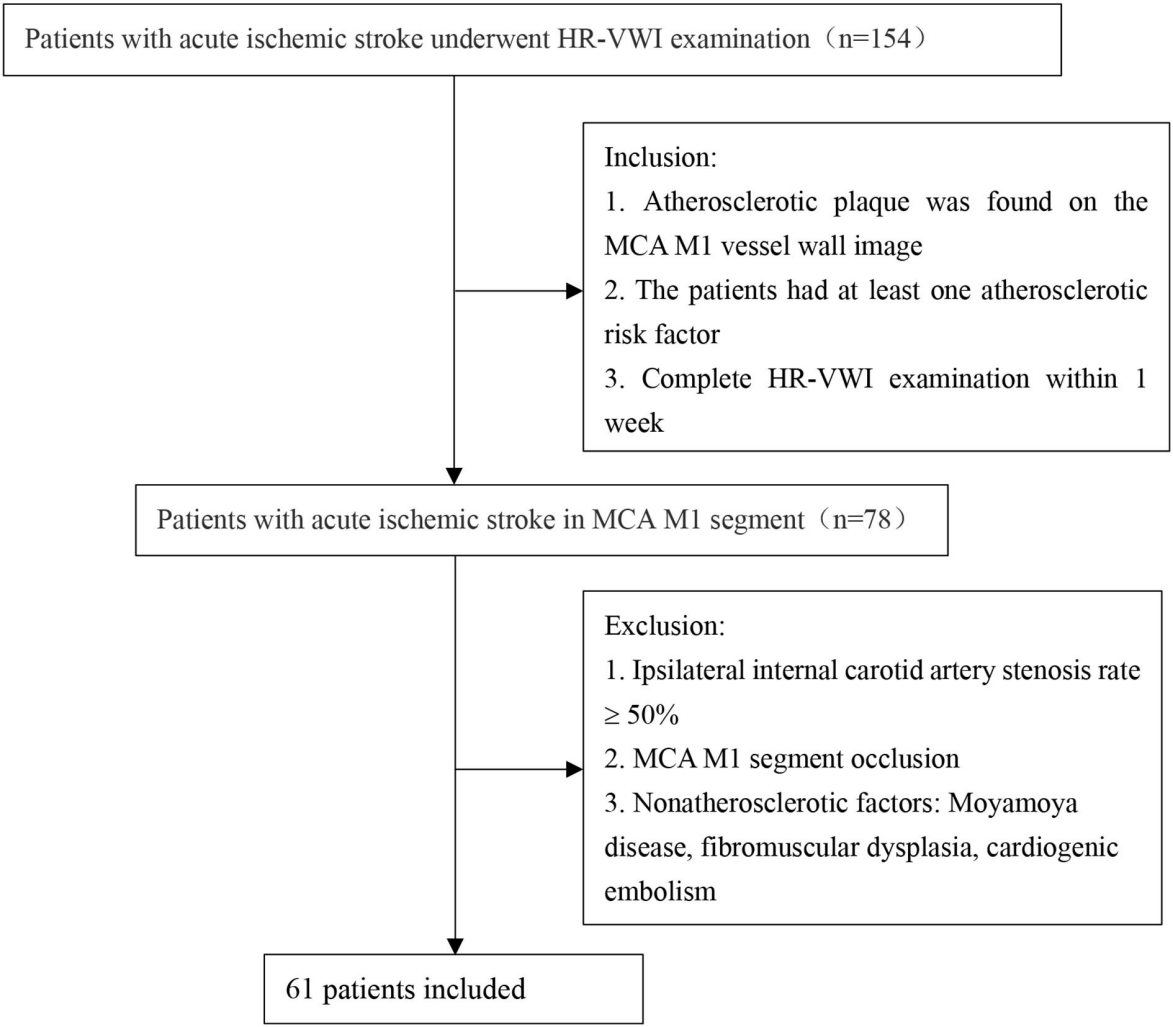
A flowchart of patient recruitment. HR-VWI: high-resolution vessel wall imaging.

The local ethics committee approved this study. From January 2019 to January 2022, we retrospectively recruited patients admitted to Shenzhen Bao'an District People's Hospital because of acute ischemic stroke caused by atherosclerosis of the M1 segment of the MCA. Acute ischemic stroke was determined based on high-signal lesions on diffusion-weighted imaging and the corresponding central nervous dysfunction in 1 week (14). The hyperintense lesions on diffusion-weighted images (DWIs) were located in the blood supply area of the middle cerebral artery. All patients underwent head magnetic resonance imaging (MRI), magnetic resonance angiography (MRA), and HR-VWI examinations within 1 week from the onset of the disease to assess intracranial atherosclerosis. We retrospectively analyzed 154 patients with acute ischemic stroke and recruited 61 patients. The specific process is shown in Figure 1. All patients satisfied the following criteria: (1) atherosclerotic plaque found on the MCA M1 vessel wall image (when there were ≥2 plaques, the plaque leading to the narrowest lumen was evaluated); and (2) the patient had at least one atherosclerotic risk factor. Patients with an ipsilateral internal carotid artery stenosis rate ≥50% and MCA M1 segment occlusion were excluded. Patients with ischemic stroke caused by nonatherosclerotic factors were also excluded.

### Classification of the probable stroke mechanisms in patients with ischemic stroke

**Figure 2:**
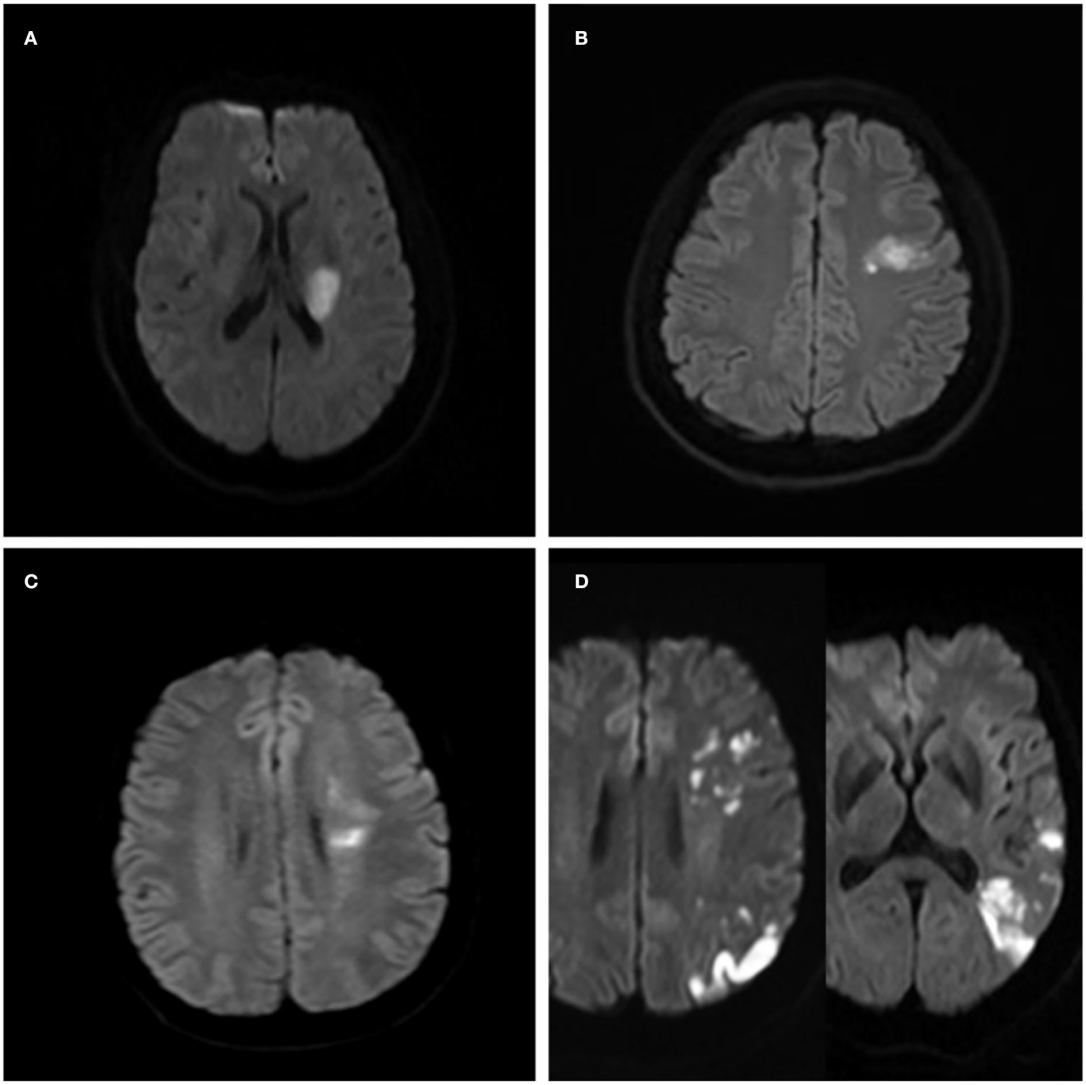
Ischemic lesion patterns indicating the different stroke mechanisms in four patients with the symptomatic middle cerebral artery (MCA) atherosclerotic stenosis. **(A)** A case of isolated acute infarction in the area of the perforating artery suggests parent artery atherosclerosis occluding a penetrating artery. **(B)** Local infarction indicating a probable artery-to-artery embolism. **(C)** Internal watershed infarctions indicating probable hypoperfusion. **(D)** Multiple cortical and wedge-shaped infarctions indicating a probable mixed mechanism of artery-to-artery embolism and hypoperfusion.

According to the China Ischemic Stroke Subclassification (CISS) and other previous studies on the mechanism of intracranial arterial stenosis (ICAS) stroke, we evaluated the distribution pattern of ischemic lesions on diffusion-weighted images (DWIs) and the location and severity of ICAS in combination with MRA. All images were analyzed with the Picture Archiving Communication System. The evidence of ischemic lesions is high-signal lesions in DWI. The possible stroke mechanisms in patients with symptomatic MCA atherosclerosis were divided into four categories (11, 15) (Figure 2). (1) Parent artery atherosclerosis occluding penetrating artery (P): the M1 segment of the MCA had any degree of stenosis, and isolated acute infarction occurred in an area adjacent to the blood supply of the perforating artery. (2) Artery-to-artery embolism (A): the presence of single or multiple small cortical infarctions, with or without subcortical infarction, or wedge-shaped infarcts, and cortical and subcortical areas that were completely located in the blood supply area of the M1 segment of the MCA but did not involve border zone areas. (3) Hypoperfusion (H): the presence of single or multiple infarcts in the watershed area, including the cortical type and the subcortical type. The cortical type refers to the area between the supplying territories of the anterior cerebral artery (ACA) and the MCA or between the MCA and the posterior cerebral artery. Infarctions in these areas are generally wedge-shaped or oval. The subcortical type refers to the white matter along and above the lateral ventricles between the deep and superficial supplying territories of the MCA or between the superficial territories of the MCA and ACA. These infarctions are linearly distributed in the centrum semiovale and the corona radiata and are present as fusion infarctions with large cigar shapes. (4) Mixed mechanisms (M): the coexistence of 2 or 3 of the mechanisms described above.

### High-resolution vessel wall imaging

A 3.0-T MRI scanner (Magnetom Skyra; Siemens, Munich, Germany) with a 20-channel phased-array head coil was used. High-resolution vessel wall scanning was performed by 3D T1-sampling perfection with application-optimized contrasts using different flip angle evolutions (SPACE), such as pre- and postcontrast scanning. This imaging sequence was applied using the following parameters: for diffusion-weighted imaging: repetition time (TR) = 4,100 ms, echo time (TE) = 64 ms, field of view (FOV) = 230 mm × 230 mm, matrix size = 160 × 160, and slice thickness = 5 mm; for time of flight: TR = 20 ms, TE = 3.69 ms, FOV = 200 × 172 mm, matrix size = 235 × 320, and slice thickness = 0.6 mm; and for 3D-T1 SPACE: TR = 980 ms, TE = 27 ms, FOV = 200 mm × 178 mm, acquired resolution = 0.78 × 0.78 × 0.78, reconstruction resolution = 0.39 × 0.39 × 0.39, and slice thickness = 0.39. Before the acquisition of the contrast-enhanced 3D-T1 SPACE sequence, 0.1 ml/kg of gadolinium-containing contrast agent (gadobutrol [Gadovist]; Bayer Pharma, Berlin, Germany) was administered to the patient (16).

### Image analysis

Plaque was defined as thickening >50% of adjacent or contralateral vessel wall thickness on both pre- and post-contrast HR-VWI (17). The plaque causing the narrowest lumen on the M1 segment of the symptomatic MCA was defined as the culprit plaque (16, 18). The HR-VWI was processed using vessel mass software (Leiden University Medical Centre, Leiden, the Netherlands), which was used to reconstruct multiple cross-sections of continuous vertical vessels with a thickness of 1 mm. Each cross-section was magnified 4-fold, and the vessel and lumen boundaries were semiautomatically tracked. These steps were repeated with the proximal end of the plaque as the reference. Four quadrants (ventral, dorsal, superior, and inferior walls) were selected at the maximal lumen narrowing (MLN) cross-section of the blood vessels. When ≥2 quadrants were involved in the plaque, the quadrant with the thickest plaque was selected. Intraplaque hemorrhage was defined as a bright T1 signal ≥ 150% of the T1 signal of the adjacent muscle or pons (19). Plaque irregularity was defined as surface underfinishing. Plaque enhancement was graded as follows: no enhancement means that in the same individual, the enhancement was similar to or smaller than the intracranial artery wall without plaque; mild enhancement means that the enhancement degree was greater than the grade intracranial artery wall but smaller than pituitary funnel; and the obvious enhancement is similar to or larger than the funnel (20). The parameters were evaluated by the MLN cross-section of blood vessels, such as the maximum wall thickness (WT_max_), lumen area (LA), outer area (OA), wall area (WA), and signals of the precontrast (Signal _pre_) and postcontrast (Signal _post_) scans of the plaque. The following formulas were used (16): plaque burden (PB) = (WA_MLN_/OA_MLN_ × 100%); enhancement ratio (ER) = (Signal _post_ – Signal _pre_)/Signal _pre_ × 100%; remodeling index (RI) = OA_MLN_/OA _ref_ (the remodeling mode is classified according to the RI value: RI ≥ 1.05 is positive remodeling (PR), RI ≤ 0.95 is negative remodeling (NR), and 0.95 < RI <1.05 is no reconstruction); and stenosis degree = (1 – LA_MLN_/LA _ref_) × 100%. All of these data were measured two times, 1 month apart, by a neuroradiologist who was unaware of the clinical details, and the averages were calculated and applied.

### Statistical analysis

This study used IBM SPSS Statistics 23.0 software for all statistical analyses. The intraobserver reliability for the measurement and evaluation of the vessel wall was determined using the intraclass correlation coefficient (ICC). Cohen's κ value was used to determine the intraobserver reproducibility. Measurement data are presented as the means ± standard deviation (SD), and counting data are presented as a percentage or frequency. Count data were analyzed using the χ^2^ test or Fisher's exact probability method. A value of *p* < 0.05 was considered statistically significant. Measurement data were analyzed using analysis of variance (ANOVA) to test differences between groups. A value of *p* < 0.05 was considered statistically significant. For the binary logistic regression analysis, we selected adjusted variables that were statistically significant in the univariate analysis.

## Results

### Clinical characteristics

In this study, 61 patients with ischemic stroke caused by atherosclerosis in the M1 segment of the MCA were included. The average age of the patients was 48.9 ± 8.4 years, and there were 47 men and 14 women. These patients were divided into different stroke mechanism subtypes. There were 23 patients in the P group, 16 patients in the A group, 12 patients in the H group, and 10 patients in the M group. Table 1 shows the baseline characteristics of the patients. There was no significant difference in the stroke risk factors between the different stroke mechanism subtypes.

**Table 1:** Baseline demographics of the patients.

**Characteristics**	**P (*n* = 23)**	**A (*n* = 16)**	**H (*n* = 12)**	**M (*n* = 10)**	***P-*values**
Male	19(82.6)	12(75.0)	9(75.0)	7(70.0)	0.841
Age, y	48.0 ± 8.0	49.3 ± 8.9	50.1 ± 7.4	48.6 ± 10.0	0.904
**Cardiovascular risk factors (%)**
Hypertension	17(73.9)	12(75.0)	7(58.3)	6(60.0)	0.673
DM	7(30.4)	2(12.5)	4(33.3)	6(60.0)	0.089
Hyperlipidemia	17(73.9)	11(68.8)	9(75.0)	7(70.0)	0.978
Smoker	17(73.9)	12(75.0)	7(58.3)	6(60.0)	0.673
**Laboratory test results (mmol/L)**
FG	6.4 ± 2.1	5.9 ± 1.6	6.2 ± 1.8	7.4 ± 1.9	0.297
TC	4.7 ± 1.2	4.8 ± 1.2	5.0 ± 1.0	4.9 ± 1.4	0.953
Triglyceride	1.6 ± 0.7	1.6 ± 1.0	1.6 ± 1.1	2.0 ± 1.2	0.720
HDL	1.1 ± 0.4	1.0 ± 0.2	1.1 ± 0.3	1.0 ± 0.2	0.724
LDL	3.0 ± 1.2	3.2 ± 0.9	3.3 ± 0.7	3.3 ± 0.9	0.748
Cysteine	14.6 ± 10.8	16.0 ± 8.6	9.9 ± 2.8	13.1 ± 4.2	0.263

DM, diabetes mellitus; FG, fasting glucose; TC, total cholesterol; HDL, high-density lipoprotein cholesterol; LDL, low-density lipoprotein cholesterol; p-values were calculated using ANOVA or the chi-squared/Fisher's exact test between the four groups, as appropriate.

### Reproducibility of the stroke mechanism classification criteria and consistency of vessel wall measurements

The intra-reader reproducibility was substantial (κ, 0.728; 95% *CI*, 0.593–0.863) for the classification of the stroke mechanisms into four categories (Table 2). When the three mechanisms existed independently without considering any other accompanying mechanisms, the intra-reader reproducibility was substantial for each mechanism. The consistencies of OA_MLN_, LA_MLN_, WA _MLN_, Signal_pre_, and Signal_post_ were excellent on a 3.0-T HR-VWI (ICC >0.75), and WT_max_, OA_ref_, and LA_ref_ were fair-to-good (0.40 < ICC <0.75).

**Table 2:** Intraobserver reproducibility of stroke mechanism classification.

	**κ (95% CI)**
Overall (4 categories)	0.728(0.593–0.863)
**Existence of each mechanism**	
P	0.827(0.972–0.682)
A	0.684(0.468–0.899)
H	0.610(0.365–0.855)

A indicates artery-to-artery embolism; H, hypoperfusion; and P, parent artery atherosclerosis occluding a penetrating artery.

### Qualitative analysis of the correlation between culprit plaque characteristics and stroke mechanism

There was a significant difference in the surface irregularity of the culprit plaques of the different subtypes of stroke mechanism (*p* = 0.001). Further pairwise comparison showed that plaque surface irregularity in the A group was more common than in the P group and the M group (*p* < 0.0125), and plaque surface irregularity in the P group was more common than in the H group (*p* < 0.0125). The difference in the T1 high signal in the culprit plaques of the different stroke mechanisms was statistically significant (*p* =0.004). Further pairwise comparison showed that a high T1 signal in the plaques of the A group was more common than in the P group (*p* < 0.0125), and a high T1 signal in the plaques of the H group was more common than in the A group and the M group (*p* < 0.0125). There was a significant difference in the pattern of culprit plaque remodeling between the different stroke subtypes (*p* =0.004). Further pairwise comparison showed that the positive remodeling in the P group was more common than in the other groups (*p* < 0.0125). There was no significant difference in the location of the culprit plaques between the different stroke subtypes (*p* = 0.061) (Table 3).

**Table 3:** Culprit plaque characteristics of the different stroke mechanism subtypes of patients with symptomatic middle cerebral artery (MCA) M1.

**Measurement**	**P (*n* = 23)**	**A (*n* = 16)**	**H (*n* = 12)**	**M (*n* = 10)**	***P-*values**
**Plaque location**					0.061
Superior	14 (60.9)	6 (37.5)	3 (25.0)	3 (30.0)	-
Ventral	3 (13.0)	6 (37.5)	3 (25.0)	49 (40.0)	-
Inferior	3 (13.0)	2 (12.5)	5 (41.7)	2 (20.0)	-
Dorsal	3 (13.0)	2 (12.5)	1 (8.30)	1 (10.0)	-
Irregularity ^a^	11 (47.8)	13 (81.3)	10 (83.3)	10 (100)	0.001
T1 hyperintensity^b^	1 (4.3)	4 (25.0)	6 (50.0)	4 (40.0)	0.004
**Remodeling mode**					0.004
PR^c^	12 (60.0)	5 (25.0)	2 (10.0)	1 (5.0)	-
NR	9 (25.7)	8 (22.9)	9 (25.7)	9 (25.7)	-
RI	1.04 ± 0.17	0.97 ± 0.16	0.86 ± 0.17	0.88 ± 0.20	0.016
**Enhancement level**					0.001
Mild	16 (72.7)	3 (13.6)	1 (4.5)	2 (9.1)	-
Obvious^d^	7 (17.9)	13 (33.3)	11 (28.2)	8 (20.5)	-
ER	0.38 ± 0.34	0.52 ± 0.26	0.62 ± 0.40	0.71 ± 0.43	0.066
WT _max_, mm	1.32 ± 0.24	1.41 ± 0.33	1.42 ± 0.21	1.44 ± 0.32	0.548
LA _MLN_, mm^2^	0.05 ± 0.02	0.03 ± 0.02	0.02 ± 0.01	0.02 ± 0.01	0.000
OA _MLN_, mm^2^	0.14 ± 0.05	0.12 ± 0.04	0.09 ± 0.03	0.09 ± 0.03	0.002
WA _MLN_, mm^2^	0.09 ± 0.03	0.08 ± 0.03	0.07 ± 0.02	0.07 ± 0.03	0.097
PB	0.65 ± 0.08	0.72 ± 0.09	0.78 ± 0.12	0.79 ± 0.06	0.000
Stenosis	0.36 ± 0.24	0.37 ± 0.24	0.58 ± 0.31	0.66 ± 0.22	0.003

The p-values were calculated using ANOVA or the chi-squared/Fisher's exact test between the four groups, as appropriate; RI indicates the remodeling index; PB, plaque burden; ER, enhancement ratio; PR, Positive remodeling; NR, Negative remodeling. (a) The A group was statistically significant compared with the other groups; (b) the A group was statistically significant compared with the P group; (c) the P group was statistically significant compared with the other groups; and (d) the A and M groups were statistically significant compared with the other groups.

### Quantitative analysis of the correlation between culprit plaque characteristics and stroke mechanism

**Figure 3:**
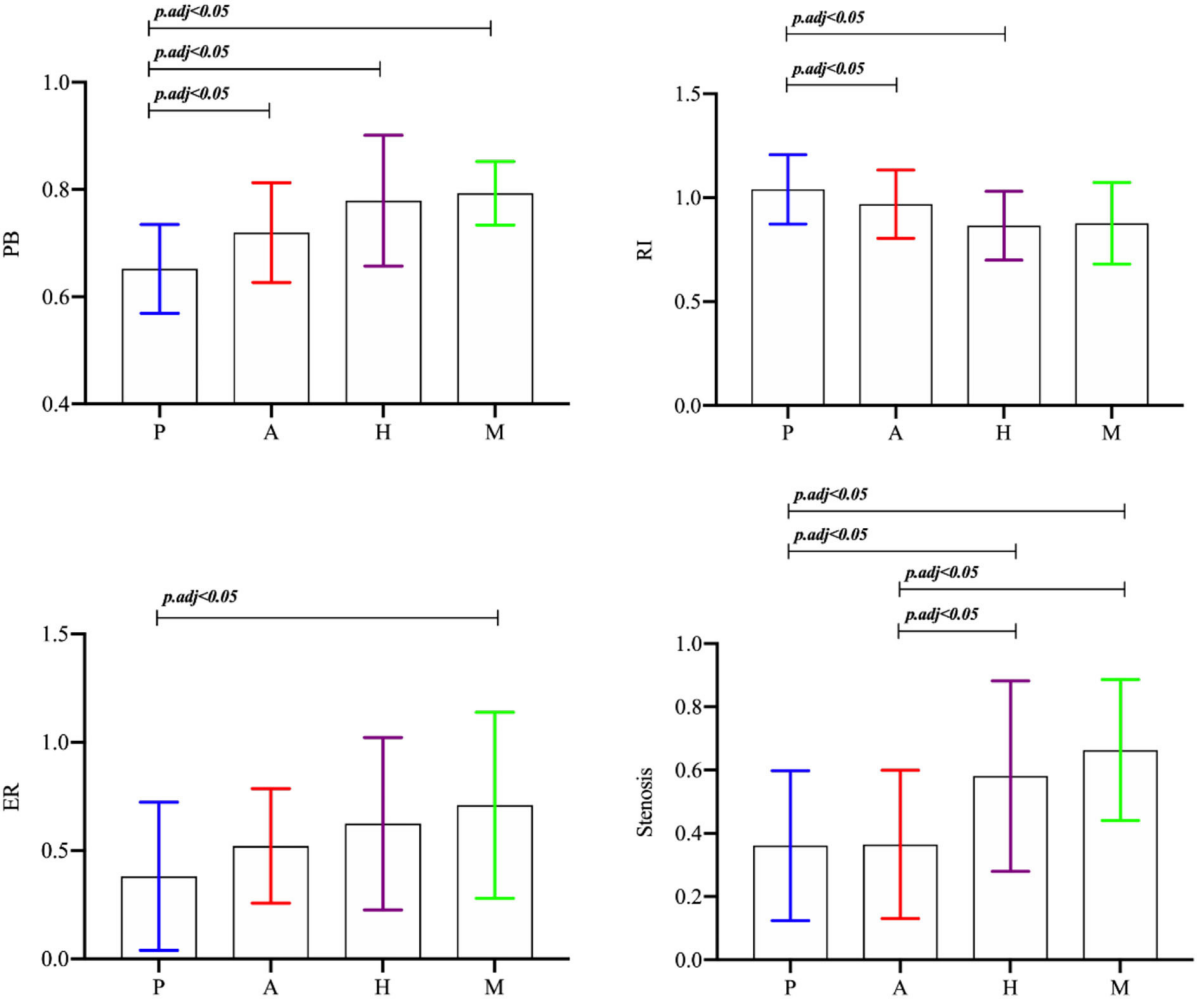
The *p-*values were calculated using ANOVA.

**Figure 4:**
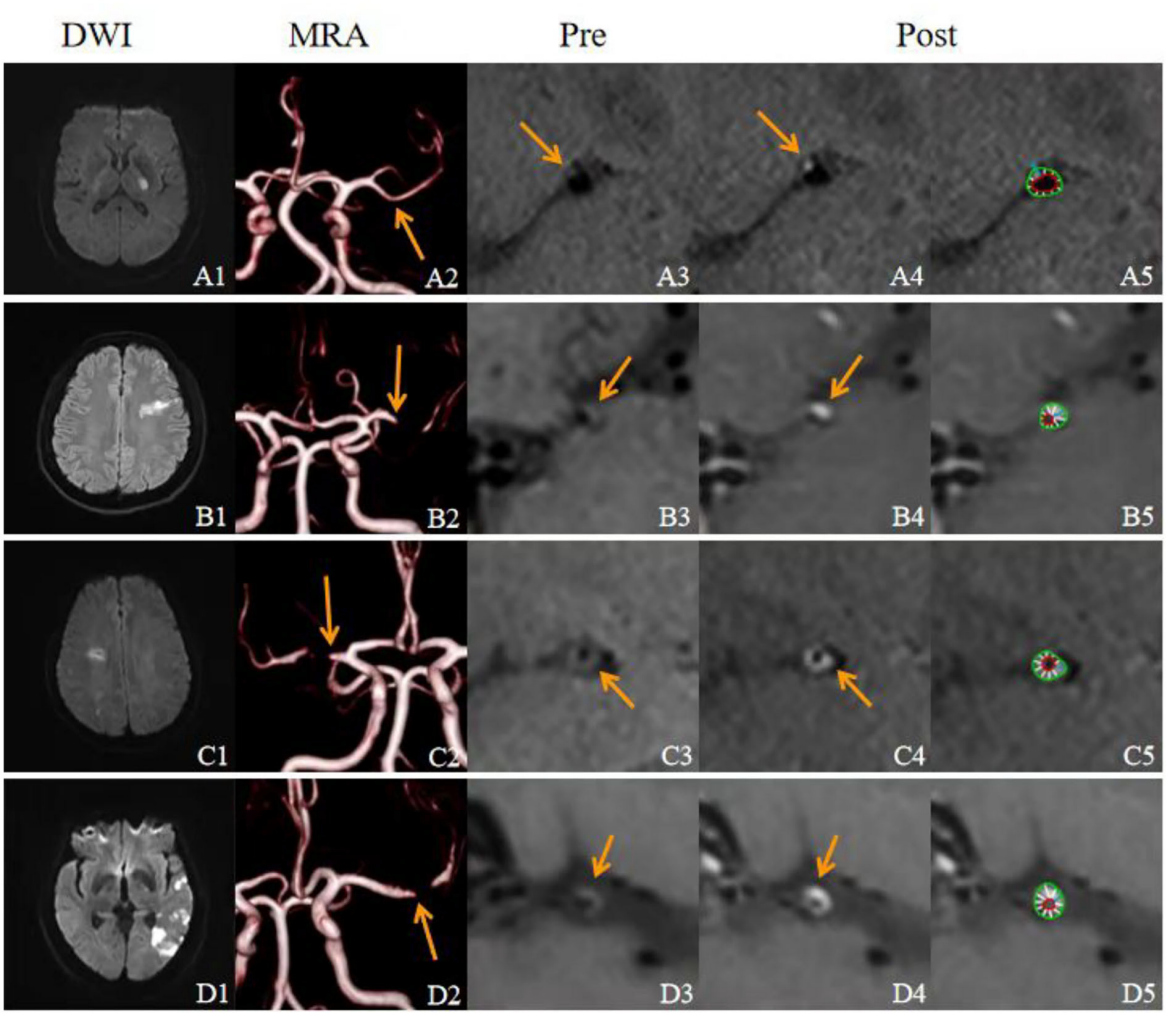
Characteristics of the culprit plaque in the M1 segment of the MCA in four patients with acute stroke with different mechanisms. **(A1–A5)**, A 39-year-old male, diffusion-weighted images (DWIs) **(A1)** showed high signal intensity in the left basal ganglia, and the stroke mechanism was parent artery atherosclerosis occluding penetrating artery by MCA atherosclerosis. Magnetic resonance angiography MRA_ **(A2)** showed that the left MCA M1 lumen was normal (arrow). Sagittal images of the precontrast **(A3)** and postcontrast **(A4)** HR-VWI showed that the plaque was located in the upper wall (arrow), showed mild enhancement, and the inner and outer walls of vessels were semiautomatically delineated on postcontrast HR-VWI **(A5)**. **(B1–B5)** A 48-year-old male, DWI **(B1)** showed multiple hypersignals in the left frontal cortex and the subcortical area, and an artery-to-artery embolism was considered the mechanism of stroke; MRA **(B2)** showed that the left MCA M1 lumen was severely stenotic (arrow); **(B3,B4)** HR-VWI showed that the plaque was located in the upper wall, which showed obvious enhancement (arrow). **(C1–C5)**, A 53-year-old male, DWI **(C1)** showed multiple hypersignals in the right subcortical watershed, and the mechanism of stroke was considered when evaluating the mechanism of hypoperfusion. MRA **(C2)** showed that the right MCA M1 lumen was severely stenotic (arrow). **(C3,C4)** HR-VWI showed that the plaque was located in the posterior wall, which showed obvious enhancement (arrow). **(D1–D5)**, A 60-year-old male, DWI **(D1)** showed multiple hypersignals in the left temporal lobe and the left posterior cortical watershed area; MRA **(D2)** showed that the left MCA M1 lumen was severely stenotic (arrow); **(D3,D4)** HR-VWI showed that the plaque was located in the posterior wall, which showed obvious enhancement (arrow).

There was no significant difference in the maximum thickness of the vessel wall between the plaques of the different stroke mechanism subtypes (*p* = 0.548). There was a significant difference in the RI between the different stroke subtypes (*p* = 0.016), and the RI in the P group was greater than in the H group and the M group (*p* < 0.0125). There was a significant difference in the PB between the different mechanisms (*p* = 0.000), and the PB in the culprit plaques was the smallest in the P group. There was a significant difference in the plaque enhancement grade between the different mechanisms (*p* = 0.001). Obvious plaque enhancement was more common in the A and M groups than in the other groups (*p* < 0.0125). The ER of the culprit plaque in the M group was higher than in the P group (*p* < 0.05). There was a significant difference in the stenosis rate of the M1 segment of the MCA between the different mechanisms (*p* = 0.003). The stenosis rate of the MCA in the H group and the M group was higher than in the P group and the A group (*p* < 0.05) (Figures 3,4).

### Association between culprit plaque characteristics and different stroke subtypes

**Figure 5:**
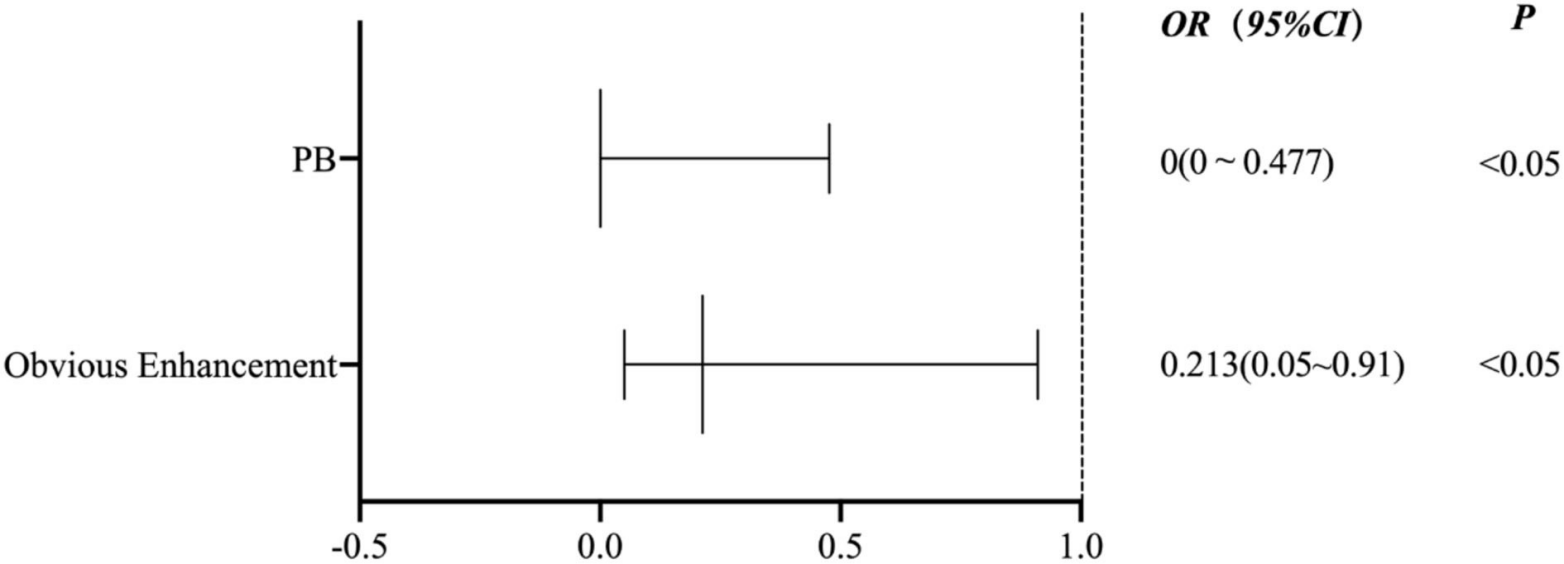
A binary logistic regression analysis results for the parameters associated with the P group compared with the other groups.

Figure 5 shows the binary logistic regression analysis results for the parameters associated with the P group compared with the other groups. After adjustment for clinical demographic factors, the enhancement level (odds ratio [*OR*] 0.213, 95% *CI* (0.05–0.91), *p* = 0.037) and the PB of culprit plaques (*OR* 0, 95% *CI* (0–0.477), *p* = 0.034) were negatively associated with the P group.

## Discussion

The present study classified the possible stroke mechanisms of MCA M1 atherosclerosis using conventional imaging (DWI and MRA). We demonstrated that there was inter-rater reproducibility of the classification criteria and correlations between the culprit plaque characteristics and different stroke mechanisms. Compared with the parent artery atherosclerosis occluding penetrating artery mechanism, ischemic stroke caused by the artery-to-artery embolism mechanism was more common and exhibited an irregular surface, high T1 signal, and obvious enhancement of the culprit plaque. Compared with the other stroke mechanisms, the PR of the culprit plaques in the P group was more common, the RI was greater, and the PB was minimal. The ER of the plaques in the P group was less than the mixed mechanism group. Compared with the artery-to-artery embolic mechanism, the T1 hyperintensity of the culprit plaque was more common in the hypoperfusion group, and the stenosis rate was greater. The binary logistic regression analysis revealed that the enhancement level (*OR* 0.213, 95% *CI* (0.05–0.91), *p* = 0.037) and PB of culprit plaque (*OR* 0, 95% *CI* (0–0.477), *p* = 0.034) were negatively associated with the P group.

Plaque enhancement may be due to gadolinium leakage caused by neovascularization, inflammation, and endothelial dysfunction. Recent studies agree that plaque enhancement is a good biomarker for intracranial atherosclerotic diseases (13), and this enhancement reflects the predictive value of ischemic stroke and may also be used as an evaluation index of curative effect. The present study showed that enhancement of the culprit plaque was more common in the artery-to-artery embolic mechanism, which indicates that the inflammatory reaction was more severe. The neovascularization in the plaque easily ruptures and bleeds, which shows a T1 high signal (21) that better indicates the instability of the plaque, and this association was confirmed by relevant pathology (22). The plaque protrudes into the lumen and causes hemodynamic changes. The low wall shear stress on the plaque surface easily induces endothelial dysfunction (23). The weakening of the fiber cap on the plaque surface increases the vulnerability of the plaque and eventually leads to plaque fragmentation. The embolus dislodges and forms an irregular shape on the surface of the plaque. The instability of plaques may affect the decision-making for clinical treatment. A study of symptomatic carotid stenosis suggested that patients treated with intravascular therapy within 2 weeks after ischemic events had a significantly higher risk of stroke or death within 30 days (26.1 vs. 1.9%) than patients treated after 2 weeks (24). The embolus is easily dislodged and may cause a distal embolism during surgery. The present study suggests that the inflammatory response to responsible plaques in the artery-to-artery embolism may be serious and should be considered. Chung et al. (25) found that high-dose statin treatment significantly reduced plaque enhancement in patients with acute stroke (*p* = 0.002). Chung et al. subsequently performed a study on statin treatment for patients with acute ischemic stroke and found that statin treatment significantly decreased the enhancement of play volume after 6 months (*p* = 0.013) (26). These studies show that high-dose statins effectively stabilize symptomatic ICAS plaques. However, the study population of Chung et al. did not include patients with artery-to-artery embolism. The Clopidogrel in High-Risk Patients with Acute Nondisabling Cerebrovascular Events (CHANCE) trial showed that the dual antiplatelet therapy of clopidogrel and aspirin effectively reduced microemboli in ischemic stroke caused by an artery-to-artery embolism (27). For patients with artery-to-artery embolic stroke, reducing the inflammatory response to the culprit plaques and the shedding of emboli may be more beneficial to patients.

In this study, we found that the PR of the culprit plaque was more common in the parent artery atherosclerosis occluding penetrating artery mechanism, the lumen stenosis rate and PB were the smallest, and the ER was lower than the mixed mechanism. Penetrating artery occlusions due to parent artery atherosclerosis are more likely associated with early neurological deterioration and recurrent stroke (28) and are classified as large artery atherosclerosis rather than small vessel occlusion (8, 29). Recent studies showed that the single subcortical infarction was primarily related to plaques of the M1 segment of the MCA located in the upper wall, and the lumen had mild stenosis or was normal (30, 31). This result is similar to the result that the culprit plaque in the perforator artery group was primarily located in the upper wall. Jiang et al. found that the plaque of a single subcortical infarct was located in the upper wall, which was related to the fewer lenticulostriate arteries (LSA) branches on the symptomatic side (*p* = 0.011) and the shorter average length (*p* = 0.025) (32). Therefore, the involved vessel wall may grow outward in the early stage of the atherosclerotic disease to compensate for the stenosis of the lumen, which results in positive remodeling and a certain degree of inflammatory response. Although the stenosis of the carrier artery lumen and the plaque burden are relatively insignificant at this time, the plaque is more likely to occlude the opening of the perforator artery, which results in a high-risk transient ischemic attack or mild ischemic stroke. Patients with minor ischemic stroke or high-risk TIA who received a combination of clopidogrel and aspirin had a lower risk of major ischemic events but a higher risk of major hemorrhage at 90 days than patients who received aspirin alone (33). The dual antiplatelet therapy of cilostazol and clopidogrel may also be better than single antiplatelet therapy (34). Cilostazol has an anti-platelet aggregation effect and dilates arterioles, which may compensate for the involvement of perforator artery openings. High-dose statin therapy improves the short-term functional prognosis (33, 35). However, the therapeutic effect on perforator artery disease must be confirmed in further randomized controlled studies. Therefore, a prospective study using statins and dual antiplatelet therapy to prevent early neurological deterioration and recurrent stroke caused by perforator atherosclerosis should be performed (10) because its efficacy is not certain.

The present study observed that a high T1 signal of the plaque with hypoperfusion mechanism was most common, and the degree of stenosis was most severe. As mentioned above, a high T1 signal reflects the inflammatory progress of plaques. However, obvious stenosis of the lumen reduces the antegrade flow to the relevant area. If the collateral circulation compensation is insufficient, the downstream perfusion may be prolonged or damaged and result in the occurrence of infarction (11, 36). Therefore, focusing only on intensive blood pressure control may increase the risk of recurrent stroke in patients with symptomatic intracranial artery stenosis with impaired perfusion (37). A *post-hoc* analysis of the Stenting and Aggressive Medical Management for Preventing Recurrent Stroke in Intracranial Stenosis (SAMMPRIS) trial also showed that among stroke patients with a stenosis rate of 70–99%, a subgroup with marginal zone infarction and collateral circulation damage had a particularly high risk of recurrent stroke despite drug treatment (38). For the stroke mechanism of hypoperfusion perfusion, the degree of inflammation of the culprit plaque and the stenosis rate of the mechanism should also be considered.

A binary logistic regression analysis revealed that the plaque enhancement level and PB were negatively associated with the parent artery atherosclerosis occluding a penetrating artery. In other words, when the enhancement of the culprit plaque and the greater plaque burden are obvious, the more likely it is to be the result of other stroke mechanisms. A recent study is similar to our results. The plaque in the branch occlusive disease group was less enhancing plaque than in the artery-to-artery group (*p* = 0.030) (39). However, there are few studies on plaque burden as an indicator of treatment evaluation. One study showed that the total volume of the carotid artery wall was significantly reduced in patients with carotid atherosclerosis with type 2 diabetes after receiving hypoglycemic drugs compared with a control group without diabetes after 2 years of treatment (40). However, the evaluation of HR-VWI related to the therapeutic effect of hypoglycemic drugs on intracranial artery atherosclerosis plaques must be further studied.

Our study has some limitations. First, it was a single-center study with relatively small sample size. Second, the study performed a retrospective analysis, and there may be a selection bias. Third, statistical analysis lacked the evaluation of normal distribution and the consistency analysis of plaque morphological characteristics. Finally, this study only recruited patients with anterior circulation stroke, and further research is needed to evaluate patients with posterior circulation stroke.

## Conclusion

Evaluations of the culprit plaque characteristics of patients with symptomatic MCA atherosclerotic in different stroke mechanisms based on HR-VWI are feasible. The plaque characteristics of different stroke mechanisms may have clinical value for the selection of treatment strategies and prevention of stroke recurrence.

## Data availability statement

The raw data supporting the conclusions of this article will be made available by the authors, without undue reservation.

## Author contributions

Conceptualization and supervision: G-hL and J-xS. Data curation: G-hL, N-xF, and T-dH. Formal analysis and writing—original draft: G-hL. Methodology: G-hL, N-xF, T-dH, and J-xS. Resources and software: J-xS. Validation: N-xF and L-lZ. Writing—review and editing: G-hL and J-xS. All authors contributed to the article and approved the submitted version.

## Conflict of interest

The authors declare that the research was conducted in the absence of any commercial or financial relationships that could be construed as a potential conflict of interest.

## Publisher's note

All claims expressed in this article are solely those of the authors and do not necessarily represent those of their affiliated organizations, or those of the publisher, the editors and the reviewers. Any product that may be evaluated in this article, or claim that may be made by its manufacturer, is not guaranteed or endorsed by the publisher.

## References

[1] Ko Y Lee S Chung JW Han MK Park JM Kang K. MRI-based algorithm for acute ischemic stroke subtype classification. J Stroke. (2014) 16:161–72. 10.5853/jos.2014.16.3.161 25328874 PMC4200592

[2] Makowski MR Botnar RM. MRI imaging of the arterial vessel wall: molecular imaging from bench to bedside. Radiology. (2013) 269:34–51. 10.1148/radiol.13102336 24062561

[3] Sato Y Hatakeyama K Marutsuka K Asada Y. Incidence of asymptomatic coronary thrombosis and plaque disruption: comparison of non-cardiac and cardiac deaths among autopsy cases. Thromb Res. (2009) 124:19–23. 10.1016/j.thromres.2008.08.026 19000633

[4] Lan L Liu H Ip V Soo Y Abrigo J Fan F. Regional high wall shear stress associated with stenosis regression in symptomatic intracranial atherosclerotic disease. Stroke. (2020) 51:3064–73. 10.1161/strokeaha.120.030615 32883193

[5] Collaborators GN. Global, regional, and national burden of neurological disorders, 1990–2016: a systematic analysis for the global burden of disease study 2016. Lancet Neurol. (2019) 18:459–80. 10.1016/s1474-4422(18)30499-x 30879893 PMC6459001

[6] Hathidara MY Saini V Malik AM. Stroke in the young: a global update. Curr Neurol Neurosci Rep. (2019) 19:91. 10.1007/s11910-019-1004-1 31768660

[7] Chen CY Lin PT Wang YH Syu RW Hsu SL Chang LH. Etiology and risk factors of intracranial hemorrhage and ischemic stroke in young adults. J Chin Med Assoc. (2021) 84:930–6. 10.1097/jcma.0000000000000598 34380990 PMC12966178

[8] Chen PH Gao S Wang YJ Xu AD Li YS Wang D. Classifying ischemic stroke, from TOAST to CISS. CNS Neurosci Ther. (2012) 18:452–6. 10.1111/j.1755-5949.2011.00292.x 22268862 PMC6493455

[9] Flusty B De Havenon A Prabhakaran S Liebeskind DS Yaghi S. Intracranial atherosclerosis treatment: past, present, and future. Stroke. (2020) 51:e49–53. 10.1161/strokeaha.119.028528 32078441 PMC7041867

[10] Huang YC Lee JD Weng HH Lin LC Tsai YH Yang JT. Statin and dual antiplatelet therapy for the prevention of early neurological deterioration and recurrent stroke in branch atheromatous disease: a protocol for a prospective single-arm study using a historical control for comparison. BMJ Open. (2021) 11:e054381. 10.1136/bmjopen-2021-054381 34836908 PMC8628334

[11] Feng X Chan KL Lan L Abrigo J Liu J Fang H. Stroke mechanisms in symptomatic intracranial atherosclerotic disease: classification and clinical implications. Stroke. (2019) 50:2692–9. 10.1161/strokeaha.119.025732 31409268

[12] Menon BK Smith EE Coutts SB Welsh DG Faber JE Goyal M. Leptomeningeal collaterals are associated with modifiable metabolic risk factors *Ann Neurol*. (2013) 74:241–8. 10.1002/ana.23906 23536377 PMC3836863

[13] Song JW Pavlou A Xiao J Kasner SE Fan Z Messé SR. Vessel wall magnetic resonance imaging biomarkers of symptomatic intracranial atherosclerosis: a meta-analysis. Stroke. (2021) 52:193–202. 10.1161/strokeaha.120.031480 33370193 PMC7773134

[14] Mendelson SJ Prabhakaran S. Diagnosis and management of transient ischemic attack and acute ischemic stroke: a review. Jama. (2021) 325:1088–98. 10.1001/jama.2020.26867 33724327

[15] Al Kasab S Derdeyn CP Guerrero WR Limaye K Shaban A Adams HP Jr. Intracranial large and medium artery atherosclerotic disease and stroke. J Stroke Cerebrovasc Dis. (2018) 27:1723–32. 10.1016/j.jstrokecerebrovasdis.2018.02.050 29602616

[16] Lin GH Song JX Fu NX Huang X Lu HX. Quantitative and qualitative analysis of atherosclerotic stenosis in the middle cerebral artery using high-resolution magnetic resonance imaging. Can Assoc Radiol J. (2021) 72:783–8. 10.1177/0846537120961312 33023323

[17] Lindenholz A Van Der Kolk AG Van Der Schaaf IC Van Der Worp HB Harteveld AA Dieleman N. Intracranial atherosclerosis assessed with 7-T MRI: evaluation of patients with ischemic stroke or transient ischemic attack. Radiology. (2020) 295:162–70. 10.1148/radiol.2020190643 32013790

[18] Xu WH Li ML Gao S Ni J Zhou LX Yao M. Plaque distribution of stenotic middle cerebral artery and its clinical relevance. Stroke, (2011, 42(10): 2957-9. 10.1161/strokeaha.111.618132 21799160

[19] Tao L Li XQ Hou X W Yang BQ Xia C Ntaios G. Intracranial atherosclerotic plaque as a potential cause of embolic stroke of undetermined source. J Am Coll Cardiol. (2021) 77:680–91. 10.1016/j.jacc.2020.12.015 33573737

[20] Qiao Y Zeiler SR Mirbagheri S Leigh R Urrutia V Wityk R. Intracranial plaque enhancement in patients with cerebrovascular events on high-spatial-resolution MR images. Radiology. (2014) 271:534–42. 10.1148/radiol.13122812 24475850 PMC4263625

[21] Derksen WJ Peeters W Van Lammeren GW Tersteeg C De Vries JP De Kleijn DP. Different stages of intraplaque hemorrhage are associated with different plaque phenotypes: a large histopathological study in 794 carotid and 276 femoral endarterectomy specimens. Atherosclerosis. (2011) 218:369–77. 10.1016/j.atherosclerosis.2011.07.104 21868015

[22] Chen XY Wong KS Lam WW Ng HK. High signal on T1 sequence of magnetic resonance imaging confirmed to be intraplaque haemorrhage by histology in middle cerebral artery. Int J Stroke. (2014) 9:E19. 10.1111/ijs.12277 24798044

[23] Leng X Lan L Ip H Abrigo J Scalzo F Liu H. Hemodynamics and stroke risk in intracranial atherosclerotic disease. Ann Neurol. (2019) 85:752–64. 10.1002/ana.25456 30840312

[24] Topakian R Strasak AM Sonnberger M Haring HP Nussbaumer K Trenkler J. Timing of stenting of symptomatic carotid stenosis is predictive of 30-day outcome. Eur J Neurol. (2007) 14:672–8. 10.1111/j.1468-1331.2007.01815.x 17539948

[25] Chung JW Hwang J Lee MJ Cha J Bang OY. Previous statin use and high-resolution magnetic resonance imaging characteristics of intracranial atherosclerotic plaque: the intensive statin treatment in acute ischemic stroke patients with intracranial atherosclerosis study. Stroke. (2016) 47:1789–96. 10.1161/strokeaha.116.013495 27301946

[26] Chung JW Cha J Lee MJ Yu IW Park MS Seo WK. Intensive statin treatment in acute ischaemic stroke patients with intracranial atherosclerosis: a high-resolution magnetic resonance imaging study (STAMINA-MRI study). J Neurol Neurosurg Psychiatry. (2020) 91:204–11. 10.1136/jnnp-2019-320893 31371644

[27] Jing J Meng X Zhao X Liu L Wang A Pan Y. Dual antiplatelet therapy in transient ischemic attack and minor stroke with different infarction patterns: subgroup analysis of the chance randomized clinical trial. JAMA Neurol. (2018) 75:711–9. 10.1001/jamaneurol.2018.0247 29582084 PMC5885215

[28] Petrone L Nannoni S Del Bene A Palumbo V Inzitari D. Branch atheromatous disease: a clinically meaningful, yet unproven concept. Cerebrovasc Dis. (2016) 41:87–95. 10.1159/000442577 26671513

[29] Gao S Wang YJ Xu AD Li YS Wang DZ. Chinese ischemic stroke subclassification. Front Neurol. (2011) 2:6. 10.3389/fneur.2011.00006 21427797 PMC3052771

[30] Sun LL Li ZH Tang WX Liu L Chang FY Zhang XB. High resolution magnetic resonance imaging in pathogenesis diagnosis of single lenticulostriate infarction with nonstenotic middle cerebral artery, a retrospective study. BMC Neurol. (2018) 18:51. 10.1186/s12883-018-1054-z 29699507 PMC5921325

[31] Shen M Gao P Zhang Q Jing L Yan H Li H. Middle cerebral artery atherosclerosis and deep subcortical infarction: a 3T magnetic resonance vessel wall imaging study. J Stroke Cerebrovasc Dis. (2018) 27:3387–92. 10.1016/j.jstrokecerebrovasdis.2018.08.013 30145026

[32] Jiang S Yan Y Yang T Zhu Q Wang C Bai X. Plaque distribution correlates with morphology of lenticulostriate arteries in single subcortical infarctions. Stroke. (2020) 51:2801–9. 10.1161/strokeaha.120.030215 32757756 PMC7447184

[33] Johnston SC Easton JD Farrant M Barsan W Conwit RA Elm JJ. Clopidogrel and aspirin in acute ischemic stroke and high-risk TIA. N Engl J Med. (2018) 379:215–25. 10.1056/NEJMoa1800410 29766750 PMC6193486

[34] Kamo H Miyamoto N Otani H Kurita N Nakajima S Ueno Y. The importance of combined antithrombotic treatment for capsular warning syndrome. J Stroke Cerebrovasc Dis. (2018) 27:3095–9. 10.1016/j.jstrokecerebrovasdis.2018.06.038 30077604

[35] Fang JX Wang EQ Wang W Liu Y Cheng G. The efficacy and safety of high-dose statins in acute phase of ischemic stroke and transient ischemic attack: a systematic review. Intern Emerg Med. (2017) 12:679–87. 10.1007/s11739-017-1650-8 28303440

[36] Nannoni S Sirimarco G Cereda C W Lambrou D Strambo D Eskandari A. Determining factors of better leptomeningeal collaterals: a study of 857 consecutive acute ischemic stroke patients. J Neurol. (2019) 266:582–8. 10.1007/s00415-018-09170-3 30610425

[37] Yamauchi H Higashi T Kagawa S Kishibe Y Takahashi M. Impaired perfusion modifies the relationship between blood pressure and stroke risk in major cerebral artery disease. J Neurol Neurosurg Psychiatry. (2013) 84:1226–32. 10.1136/jnnp-2013-305159 23933741 PMC3812848

[38] Wabnitz AM Derdeyn CP Fiorella DJ Lynn MJ Cotsonis GA Liebeskind DS. Hemodynamic markers in the anterior circulation as predictors of recurrent stroke in patients with intracranial stenosis. Stroke. (2018) 56:20840. 10.1161/strokeaha.118.020840 30580705 PMC6559874

[39] Won SY Cha J Choi HS Kim YD Nam H S Heo JH. High-resolution intracranial vessel wall MRI findings among different middle cerebral artery territory infarction types. Korean J Radiol. (2022) 23:333–42. 10.3348/kjr.2021.0615 35213096 PMC8876648

[40] Strang AC Van Wijk DF Mutsaerts HJ Stroes ES Nederveen AJ Rotmans JI. Guideline treatment results in regression of atherosclerosis in type 2 diabetes mellitus. Diab Vasc Dis Res. (2015) 12:126–32. 10.1177/1479164114559511 25589481

